# Solving text clustering problem using a memetic differential evolution algorithm

**DOI:** 10.1371/journal.pone.0232816

**Published:** 2020-06-11

**Authors:** Hossam M. J. Mustafa, Masri Ayob, Dheeb Albashish, Sawsan Abu-Taleb

**Affiliations:** 1 Data Mining and Optimization Research Group, Center of Artificial Intelligence Technology, Faculty of Information Science and Technology, University Kebangsaan Malaysia, Bangi, Malaysia; 2 Computer Science Department, Prince Abdullah bin Ghazi Faculty of Information and Communication Technology, Al-Balqa Applied University, Salt, Jordan; Universiti Sains Malaysia, MALAYSIA

## Abstract

The text clustering is considered as one of the most effective text document analysis methods, which is applied to cluster documents as a consequence of the expanded big data and online information. Based on the review of the related work of the text clustering algorithms, these algorithms achieved reasonable clustering results for some datasets, while they failed on a wide variety of benchmark datasets. Furthermore, the performance of these algorithms was not robust due to the inefficient balance between the exploitation and exploration capabilities of the clustering algorithm. Accordingly, this research proposes a Memetic Differential Evolution algorithm (MDETC) to solve the text clustering problem, which aims to address the effect of the hybridization between the differential evolution (DE) mutation strategy with the memetic algorithm (MA). This hybridization intends to enhance the quality of text clustering and improve the exploitation and exploration capabilities of the algorithm. Our experimental results based on six standard text clustering benchmark datasets (i.e. the Laboratory of Computational Intelligence (LABIC)) have shown that the MDETC algorithm outperformed other compared clustering algorithms based on AUC metric, F-measure, and the statistical analysis. Furthermore, the MDETC is compared with the state of art text clustering algorithms and obtained almost the best results for the standard benchmark datasets.

## Introduction

Data clustering is a common data mining task that has been applied in several applications to understand the hidden structures in data. It is considered as an essential task in several disciplines such as Information Retrieval [1], Internet of Things [2], Image segmentation [3], and wireless sensor networks [4]. Moreover, one of the widespread applications of data clustering is text clustering (TC), which is considered as an unsupervised learning method that operates without the prior knowledge of the text document labels [5]. The text clustering is utilized to cluster a vast quantity of disordered text documents as a result of the expanded big data and online information [6,7]. Thus, the text clustering aims to group a collection of text documents into a group of clusters according to the related contents and topics. A particular cluster may include all related documents, and other clusters include irrelevant documents [8–10].

Recently, many researchers used the metaheuristic algorithms to address the text clustering problem [6,8], such as krill herd algorithm (KHA) [10], particle swarm optimization (PSO) [11]. The trade-off between exploration and exploitation in these algorithms plays a vital role in improving the performance of the clustering algorithm, which can be enhanced to seek reasonable clustering solutions based on specific datasets [12,13]. However, some algorithms were unable to find robust and effective results across many datasets [10]. This may occur due to an inefficient balance between exploitation and exploration that may lead to stagnation or premature convergence [14]. Some recent studies have suggested hybridizing a local search and a global search to obtain a good balance. The local search manages the exploitation, whereas the global search manages the exploration [10,15–17].

Moreover, the optimization framework of the Memetic Algorithms (MAs) can utilize the strength of different optimization algorithms by hybridizing them within the MA framework, which may offer better performance [18]. The MA includes several evolutionary steps that help in solving many complex optimization problems [18–21]. Consequently, the MA can be hybridized with the Differential Evolution algorithm (DE), which revealed good performance over several optimization problems.

Therefore, in this work, we propose a memetic differential evolution algorithm to address the text clustering. The offered MDETC algorithm employs the approach of hybridization between the Differential Evolution and the Memetic algorithms to address the text clustering problem. The purposed text clustering algorithm aims to produce high-quality clustering measures such as AUC metric and F-measure.

### Related work

The primary task of text clustering is to group sets of documents into homogeneous clusters [28]. This task can be achieved by employing a suitable similarity function that should be maximized/minimized the similarity between the documents clusters [6]. Several researchers have used metaheuristic optimization algorithms to solve the text clustering problem such as Genetic Algorithm [22,23], Particle Swarm Optimizer algorithm [24,25], Cuckoo search [26], Ant colony optimization [27], Artificial bee colony algorithm [28,29], Firefly algorithm [30], Harmony Search [31], and the hybrid metaheuristic approaches [32–37].

Some studies employed the Genetic Algorithm to address the text clustering problem. For example, [22] proposed a text clustering method based on Genetic Algorithm that employed the ontology and the thesaurus using several similarity measures. The researchers in [23] introduced a text clustering method based on Genetic Algorithm, which was utilized separately to every cluster to avoid the local optima. Moreover, the Particle Swarm Optimizer algorithm used in some studies to reach an optimal solution. For example, [24] offered a hybridized Particle Swarm Optimizer with a k-mean algorithm with benchmark text datasets. The authors of [25] proposed text clustering based on the Particle Swarm Optimizer algorithm (EPSO). Their algorithm seeks a multi-local optimal solution using the Particle Swarm Optimizer.

Additionally, the researchers used the Cuckoo search to address the text clustering problem. For example, [26] introduced a data clustering method based on and fuzzy cuckoo optimization algorithm (FCOA) and the cuckoo optimization algorithm (COA). Other metaheuristics such as Ant colony optimization utilized to solve the text clustering problem, for example, [27] proposed a document clustering algorithm based on Ant colony optimization. Thus, the Artificial bee colony algorithm utilized to improve the text document clustering algorithm, for example, [28] employed the chaotic map model in the local search to improve the exploitation capability of the Artificial bee colony. The study of [29] utilized the Artificial bee colony algorithm in the text document clustering using the gradient search and the chaotic local search to enhance the exploitation capability of the Artificial bee colony.

Moreover, the Firefly algorithm (FA) used in [30] to address dynamic text document clustering using a Gravity Firefly Clustering (GF-CLUST). Other studies utilized the Harmony Search for the text document clustering. For example, [31] introduced the factorization approach to enhance the text document clustering.

The hybrid metaheuristic approaches are used to address the text clustering problem. For example, [32] combined Particle Swarm Optimizer with the Genetic Algorithm to address the text clustering problem. Their algorithm employed a Genetic Algorithm to enhance the global search and the Particle Swarm Optimizer to produce the range of search space. The researchers in [33] proposed a text clustering algorithm based on the combination between Particle Swarm Optimizer and Cuckoo search algorithm. Many other studies utilized the metaheuristic optimization algorithms to avoid the local optima problem of the K-means algorithm. For example, [34] applied the Harmony Search algorithm with text clustering to seek optimal clustering. Their proposed algorithm hybridized Harmony Search using the local search within the k-mean algorithm. The research of [35] hybridized k-mean with the Cuckoo search (CS) algorithm for addressing the web document clustering. The hybridization aims to improve the performance of web search results. The authors of [36] proposed a hybrid optimization algorithm to address the data clustering problem. Their algorithm combined the k-mean algorithm with the Tabu search (TS) to avoid the local optima problem. The researcher in [37] combined the Firefly algorithm with the k-mean algorithm. In their proposed algorithm, the Firefly algorithm employed to seek optimal centroids of the clusters that initialize the k-mean algorithm.

Other studies used the memetic differential evolution approach to solve several data clustering problems. For example, the study of [21] introduced a memetic differential evolution algorithm for solving data clustering problems. The algorithm proposes a clustering algorithm based on a modified adaptive Differential Evolution mutation algorithm and a local search algorithm to enhance the balance between exploration and exploitation. The experiments were based on several low dimensional real-life benchmark datasets obtained from the UCI repository of the machine learning databases. Additionally, the research employed the intra-cluster distance with the Euclidian distance similarity/dissimilarity function. Despite that this method was an effective approach to find reasonable clustering solutions, it may fail to find better solutions for high dimensional datasets such as text clustering problems. This may occur due to the utilization of inappropriate objective function that may lead to an imbalance between exploration and exploitation for high dimensional datasets.

Despite that the methods of the text clustering algorithms based on several metaheuristics approaches have better performance than other earlier algorithms, the problem of weak convergence exists in many metaheuristics algorithms. Specifically, the exploration and exploitation trade-off of the metaheuristics algorithms can be further enhanced.

### Contribution of this paper

This paper aims to tackle the issues discussed above, which can help in solving the text clustering problem by using a memetic differential evolution algorithm. More precisely, our contribution significance is two-fold.

1. We introduced a memetic Differential Evolution algorithm to address the text clustering problem. The introduced text clustering algorithm combined the MA and DE algorithms to solve the text clustering problem.
2. We developed a modified DE Mutation phase that can be applied to enhance the search of the text clustering algorithm.

More specifically, the proposed text clustering algorithm utilizes a DE mutation that is coupled with the memetic algorithm evolutionary steps. The mutation step intends to improve the search abilities of DE by employing an adaptive mutation strategy. Moreover, the improvement phase is modified to remove the duplicated solutions, which aim to avoid falling into premature convergence. The restart phase was modified by replacing a portion of the population with new solutions that are randomly generated to improve the diversity of the population.

### The organization of the paper

This paper contains the following sections: The second section presents the concepts and background such as text clustering, DE and MA. In the third section, discusses the proposed memetic DE for the text clustering problem. The fourth section discusses the results of the MDETC algorithm experiments. Lastly, the fifth section discusses the conclusions and future works of the research.

## Background

This section presents the necessary concepts of text clustering problem, memetic algorithm, and differential evolution (DE) algorithm, which are employed in the offered data text clustering algorithm.

### Text clustering problem

Text document clustering is a method of splitting a set of *n* text documents into a group of *K* clusters, which can be grouped using a particular dissimilarity/similarity measure. The *n* text documents are denoted by a set *D* = {*d1*, *d2*, …, *dn*}, the *K* clusters are represented by *C* = {*C1*, *C2*, …, *CK*}, where the entire text documents in each cluster are similar, and other text documents are dissimilar. Thus, the number of clusters is given in advance [10,38].

The pre-processing steps of the text should be used to decrease the number of text attributes/features to support the algorithm task. The pre-processing steps are organized into (a) Tokenization (b) Removal stop word (c) Stemming (d) Feature selection and (e) Calculate the terms weighing [10]. The text documents can be represented by the vector space model (*VSM*) as presented in Eq (1). *VSM* model denotes each document *i* as a vector of length *t* [10].

$$VSM=\left[\begin{array}{ccccc} w_{1,1} & w_{1,2} & \cdots & w_{1,(t-1)} & w_{1,(t)}\\ \ldots & \ldots & \ldots & \ldots & \ldots \\ \vdots & \vdots & w_{i,j} & \vdots & \vdots \\ w_{(n-1),1} & w_{(n-1),2} & \cdots & \cdots & w_{(n-1),t}\\ w_{n,1} & w_{n,2} & \ldots & w_{n,(t-1)} & w_{n,t} \end{array}\right]$$ (1)

The *wi*,*j* denotes the value of the *tf/idf* weight of term *j* in document *I*, which is commonly used term weighting method that measures whether the term is frequent or rare across all documents [24], and calculated using Eq (2). The *tf (i*,*j)* denotes the frequency of term *j* in document *i*, and *n* denotes the total number of documents in *D*, the *df (j)* is term *j* frequency in all documents [10]:

$$w_{i,j}=tf\left(i,j\right)\times \log\left(\frac{n}{df\left(j\right)}\right)$$ (2)

The text document clustering problem can be formulated in Eq (3):

$$\begin{array}{l} Optimize\\ \;C \end{array}\;f\left(D,C\right)$$ (3)

The *f(D*, *C)* represents the fitness function that measures the quality of the clusters that is produced by the text clustering methods. Hence, the fitness function can be minimized or maximized subject to the employed dissimilarity/similarity measure. The quality of the text clustering solutions can be measured by the intra-cluster distance dissimilarity/similarity measure, which is commonly utilized in text clustering [10], as shown in the Eq (4):

$$f\left(D,C\right)=\sum_{l=1}^{k}\sum_{di\in Cl}^{n}d\left(d_{i},Z_{l}\right)$$ (4)

The *d(di*, *Zl)* denotes the distance between the centroid of cluster *Zl* and text document *di*. The cosine distance is one of the most widely used distance functions in text clustering [10,38]. It can measure the similarity between document *di* and the centroid of cluster *Zl* inside the same cluster, as in Eq (5).

$$d_{\mathrm{cosine}}\left(d_{i},Z_{l}\right)=\frac{\sum_{j=1}^{t}w\left(d_{i},t_{j}\right)\times w\left(Z_{l},t_{j}\right)}{\sqrt{\sum_{j=1}^{t}w{\left(d_{i},t_{j}\right)}^{2}}\sqrt{\sum_{j=1}^{t}w{\left(Z_{l},t_{j}\right)}^{2}}}$$ (5)

The *w(Zl*, *tj)* denotes the weight of term *j* in the centroid number *l*, *and w(di*, *tj)* denotes the weight of term *j* in document *i*. Additionally, centroids *Zl* can be manipulated as the average value of the entire cluster text documents, as shown in Eq (6). The *nl* denotes the number of text documents in cluster *Zl*.

$$Z_{l}=\frac{1}{n_{l}}\sum_{\forall O_{i}\in Z_{l}}\left(d_{i}\right)$$ (6)

### Differential evolution algorithm

The Differential Evolution algorithm (DE) is considered as an effective metaheuristic evolutionary algorithm that was introduced to solve continuous and combinatorial optimization problems [19]. DE begins by population initialization. At every iteration, parents are chosen from solutions for the crossover and mutation, to produce the trial solution [19]. The mutation phase is responsible for perturbing the solution by a scaled differential vector, which includes many randomly chosen solutions to generate the mutant solution. The parent solutions are compared with the offspring solution utilizing the fitness function; the better one is then selected as the new solution to the subsequent iteration. The algorithm terminates when a condition is met, and the problem’s solution is chosen as the best individual in the population.

### Memetic algorithms

Memetic Algorithm (MA) is a metaheuristic algorithm that combines the problem-specific solvers with the evolutionary algorithm. The solvers can be performed as an approximation, local or exact search heuristics. The combination intends to find better solutions and find unreachable solutions by the local search methods or the evolutionary algorithms alone. Besides, MAs provide an optimization framework that integrates various local search strategies, learning strategies [39], perturbation mechanisms, and population management strategies [40]. MAs have several names in the literature, such as Lamarckian EA, hybrid Genetic Algorithm, or Baldwinian evolutionary algorithm.

The MA utilized other optimization algorithms by employing them inside the framework [41]. For example, metaheuristic algorithms such as Differential Evolution has shown better mutation performance [42] with appropriate parameter settings and mutation strategies. The combination of DE within the MA can offer three benefits: Firstly, the offspring’s quality produced by evolutionary algorithms such as MA can be improved by implementing several search methods in the optimization search process. An example of these search methods is the DE mutation, which can be employed to generate better quality individuals [43]. Secondly, premature convergence and stagnation can be minimized when employing a DE algorithm by balancing exploitation and exploration, which can be achieved by utilizing several mutation strategies [19]. Thirdly, the DE population can stagnate when the offspring are less fit than their parents over a given number of iterations. To address this, the DE’s performance can be enhanced by employing a convenient hybridization including local search algorithms within the MA framework [43,44].

The Memetic Algorithm includes the initialization procedure that creates solutions of the initial population; the compete procedure that is utilized to reconstruct the current population using the previous population, and the restart procedure, which is started on every degenerate state of the population [45].

### Proposed algorithm

This section describes the evolutionary steps and the solution representation of the introduced MDETC algorithm.

### Solution representation

**Fig 1:**
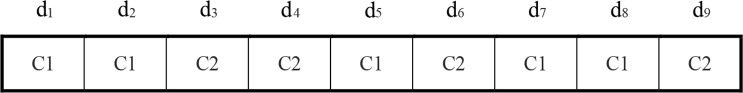
Example of the label-based representation of a candidate solution.

The label-based solution representation is employed to represents the candidate solution in the text clustering problem. Each solution represents a set of *n* documents that contain the cluster number related to each document. Fig 1 shows an example of the label-based representation of a candidate solution that contains two clusters and nine documents.

**Fig 2:**
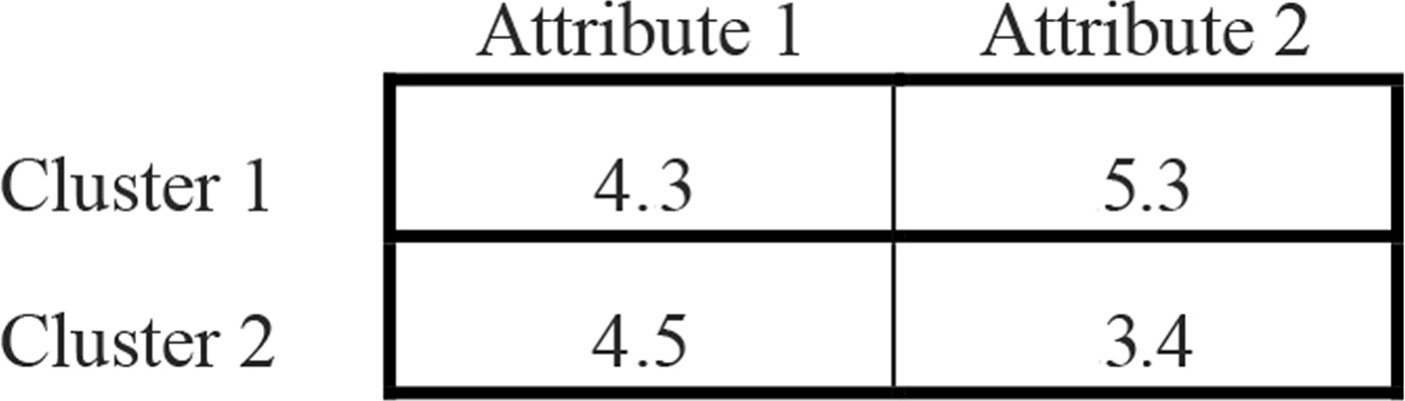
Example of the centroid-based representation of a candidate solution.

Moreover, a centroid-based 2-dimensional array is employed in the local search to store the centroid values of the clusters. The array includes *D* columns and *K* rows, where the total number of the attributes is denoted by *D*, and *K* is the number of the clusters. Fig 2 presents an example of a candidate solution of a dataset that contains two attributes and two clusters.

### The MDETC proposed algorithm

**Fig 3:**
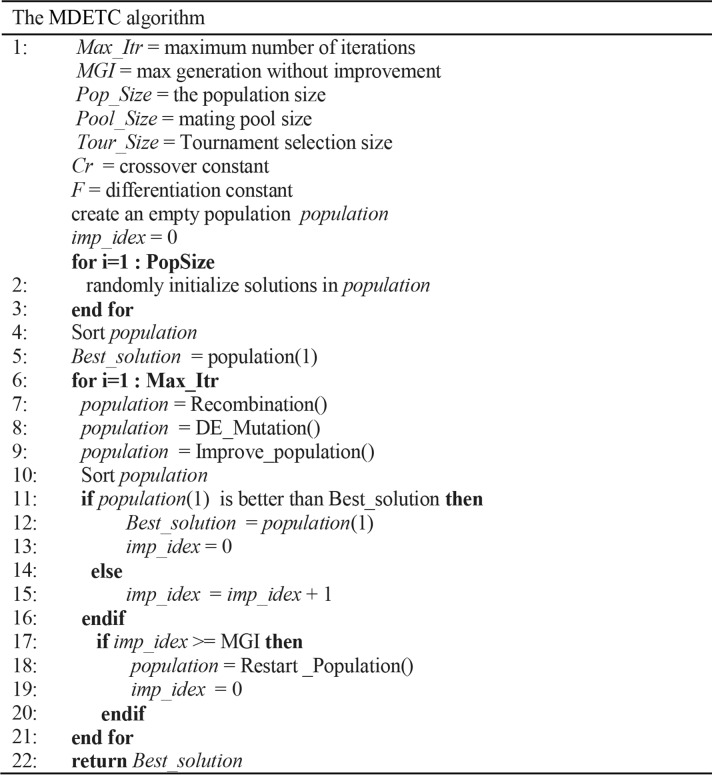
The pseudo-code of the proposed MDETC algorithm.

In MDETC, the DE mutation is hybridized with the evolutionary steps of the MA that utilizes an adaptive strategy DE/current-to-best/1. The hybridization aims to improve the convergence rate. Thus, premature convergence can be prevented in the restart step by rebuilding the diversity of the population. At last, the improvement step plays an important role to seek better solutions. The pseudo-code for the proposed MDETC algorithm is presented in Fig 3, which consist of the following phases:

#### The population initialization phase

The initial solutions of MDETC are randomly generated. The documents are grouped into *K* random clusters; every cluster’s centroid is computed by Eq (6). These steps are repeated to produce *Pop_Size* random solutions.

#### The recombination phase

The mating pool approach [46] is employed in this phase with a size of *Pool_Size*. This phase also employs the tournament selection with a size of *Tour_Size* [47], which is combined with the mating pool. The two-point crossover is then applied to the mating pool. At last, the population is joined with the mating pool, where the worst individuals in the population are replaced with new individuals from the mating pool.

#### The DE mutation phase

**Fig 4:**
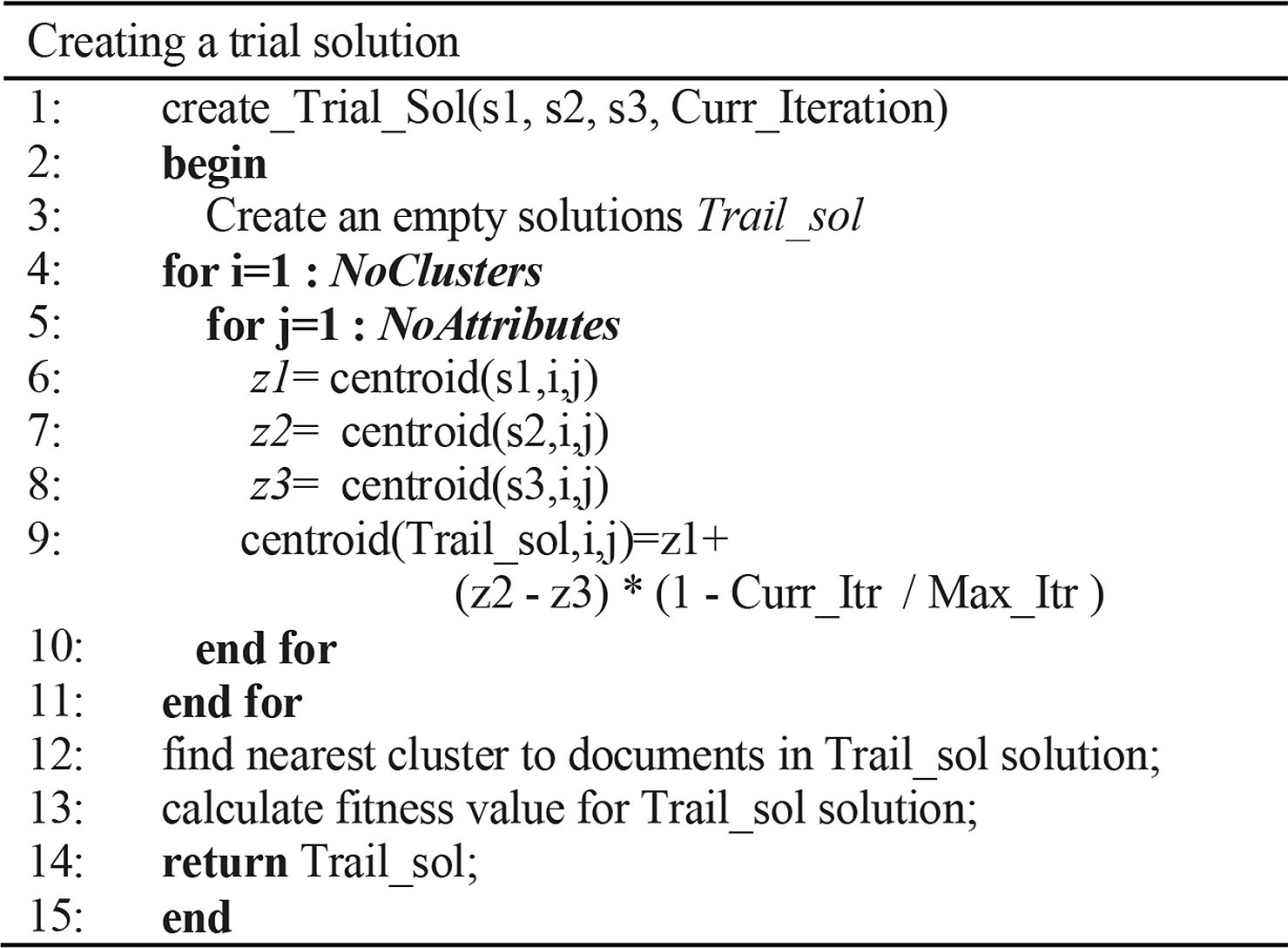
The pseudo-code of creating a trial individual algorithm.

This phase utilizes the *DE/current-to-best/1* strategy [21], as shown in Fig 3. The cluster centroids are adjusted in the mutation step to obtain better solutions, as presented in Fig 4. This is accomplished with Eq (7). The *Zbest* is the best solution centroid, *Zi* denotes the current solution centroid, *Zrand* denotes a random centroid, the *Curr_Iteration* denotes the current MDETC algorithm iteration number, and *Max_Iterations* is the maximum number of iterations of MDETC.

$$Z_{i}=Z_{current}+\left(\left(Z_{best}-Z_{rand}\right)\times \left(1-\frac{Curr\_Iteration}{Max\_Iterations}\right)\right)$$ (7)

#### The improvement phase

The improvements step clears the duplicated solutions, which guarantees better diversity in the population to prevent any premature convergence.

#### The restart phase

Whenever the population falls into the degeneration state, the restart step is invoked [45]. The restart strategy retains some portion of the population and excepts the other solutions by generating new solutions. The MDETC preserve 75% of the population for the subsequent iteration, while the rest of the population is produced randomly.

## Experimental results and setup

### Experimental setup

The MDETC performance is studied using six standard real datasets from the Laboratory of Computational Intelligence (LABIC) and represented in numerical form after the extraction of the terms. These datasets contain different variety of characteristics, such as the number of terms, clusters, and documents, and variety of complexity [48], where the datasets that been used are CSTR, tr41, tr23, tr12, tr11, and oh15, as shown in Table 1. To assess the efficiency of the introduced algorithm, the performance of MDETC is compared with the K-means algorithm, DE [21], and Genetic Algorithm (GA) [22], where the algorithms are implemented using the same experimental setup.

**Table 1:** The characteristics of the used LABIC datasets.

Dataset	Source	No. of documents	No. of terms	No. of clusters
CSTR	Technical Reports	299	1725	4
tr41	TREC	878	7454	10
tr12	TREC	313	5804	8
tr23	TREC	204	5832	6
tr11	TREC	414	6429	9
oh15	MEDLINE	913	3100	10

The algorithms’ performance is evaluated using the F-measure, which matches the ground truth with the obtained clustering solution to identify the correspondence between them. Also, the receiver operating characteristic curves (ROC) are plotted and the area under the curve (AUC) metric was calculated. A higher value of the AUC metric and F-measure means better quality of the clustering algorithm, which both range from 0 to 1.

The ROC curve can measure the degree of separability, which shows the capability of the algorithm to distinguish between classes. The ROC curves are plotted using the True Positive Percentage (TPP) against the False Positive Percentage (FPP). The TPP and FPP are computed using Eq (8) and Eq (9).

$$\mathrm{TPP}=\frac{\mathrm{Number}\;\mathrm{of}\;\mathrm{true}\;\mathrm{positives}}{\mathrm{Number}\;\mathrm{of}\;\mathrm{true}\;\mathrm{positives}+\mathrm{Number}\;\mathrm{of}\;\mathrm{false}\;\mathrm{negatives}}$$ (8)

$$\mathrm{FPP}=\frac{\mathrm{Number}\;\mathrm{of}\;\mathrm{false}\;\mathrm{positives}}{\mathrm{Number}\;\mathrm{of}\;\mathrm{true}\;\mathrm{negatives}+\mathrm{Number}\;\mathrm{of}\;\mathrm{false}\;\mathrm{positives}}$$ (9)

The F-measure of cluster *S*_j_ can be computed using the recall and precision, which are shown in Eq (10) and Eq (11), Where *Nij* denoted the number of objects of class *Ci* in cluster *Sj*, |*Sj*| is the number of objects in cluster S*j*, and *|Ci|* is the number of objects in class *Ci*. The F-measure is computed using Eq (12).

$$recall\left(C_{i},S_{j}\right)=\frac{N_{ij}}{\left|C_{i}\right|}$$ (10)

$$precision\left(C_{i},S_{j}\right)=\frac{N_{ij}}{\left|S_{j}\right|}$$ (11)

$$F-measure\left(C_{i},S_{j}\right)=\frac{2\times precision\left(C_{i},S_{j}\right)\times recall\left(C_{i},S_{j}\right)}{precision\left(C_{i},S_{j}\right)+recall\left(C_{i},S_{j}\right)}$$ (12)

The settings of the parameters of the MDETC algorithm were separately tested 31 times on all datasets; the average values of the AUC metric and F-measure were calculated. The parameter setting of the proposed MDETC is shown in Table 2, which is based on an experimental basis and the drawing on previous work from the scientific literature [21]. At last, the algorithms are applied using Oracle Java 1.8, where it was run on a personal computer with an Intel Core i7 CPU (2.6GHz) and a RAM of 8 GB size.

**Table 2:** Parameters setting used in experiments.

parameter	Value
No. of generations	100
Population size	20
Tournament selection size	10
Recombination mating pool size	10
Max Gen without improve	20
Crossover probability	0.9
DE mutation scaling factor	0.7

## Experimental results and discussion

Table 3 shows the average results of the AUC metric obtained by MDETC and the competing algorithms. The proposed MDETC achieved the best results on tr23, tr12, tr41, CSTR, and oh15 datasets, also it achieved the second-best result on the tr11 dataset. Based on AUC metric results, the MDETC obtained an excellent performance on tr41, CSTR, and oh15 datasets. Besides, MDETC obtained fair performance on tr23, tr11, and tr12 datasets. The results show that the proposed MDETC algorithm has a higher AUC metric compared with the competing algorithms, for example, the results of tr41 dataset indicates that MDETC obtained an AUC metric value of 0.9511, whereas the F-measure results of K-means, DE, and GA are 0.5533, 0.5081, and 0.49, respectively.

**Table 3:** The comparison of AUC values obtained by the MDETC, K-means, DE and GA algorithms.

Dataset	K-means	DE	GA	MDETC
tr23	0.4697	0.5	0.4457	0.**5575**
tr11	0.4745	0.4701	**0.5212**	0.5206
tr12	0.4259	0.4438	0.4524	**0.4577**
tr41	0.5533	0.5081	0.49	**0.9511**
CSTR	0.5555	0.5706	0.5337	**0.802**
oh15	0.5335	0.5588	0.5635	**0.9052**

Moreover, the results in Table 3 are further analyzed using the rankings generated by Friedman’s test based on the AUC metric, as shown in Table 4. Friedman’s test has shown that MDETC obtained a significant difference with a *p*-value of 0.03207 that is below the significance level (*α* = 0.05). The results confirm that MDETC obtained the best ranking based on the AUC metric. The DE obtained the second-best rank, and then the GA algorithm. Finally, K-means achieved the worst rank.

**Table 4:** Friedman test ranking for MDETC, K-means, DE and GA algorithms based on the AUC metric.

Algorithm	Ranking
MDETC	1.1666
DE	2.8333
GA	2.8333
K-means	3.1666

Moreover, the statistical difference between the control case (MDETC) and the other algorithms is detected using the Holm’s post-hoc procedure. Table 5 demonstrates the *p*-value achieved by Holm’s procedure, where the null hypothesis is rejected based on the achieved *p*-value that needs to be less than the adjusted value of α (α*/i*). The value of *i* represents the rank of each algorithm. The Holm’s procedure demonstrates that MDETC is statistically better than K-means, DE and GA based on the AUC metric.

**Table 5:** Comparison between MDETC, K-means, DE and GA algorithms using Holm’s post-hoc procedure based on the AUC metric.

*i*	Algorithm	α/*i*	*p*-value of Holms	Null Hypothesis
1	DE	0.05/1 = 0.0500	0.02534	Rejected
2	GA	0.05/2 = 0.0250	0.02434	Rejected
3	K-means	0.05/3 = 0.0166	0.00729	Rejected

**Fig 5:**
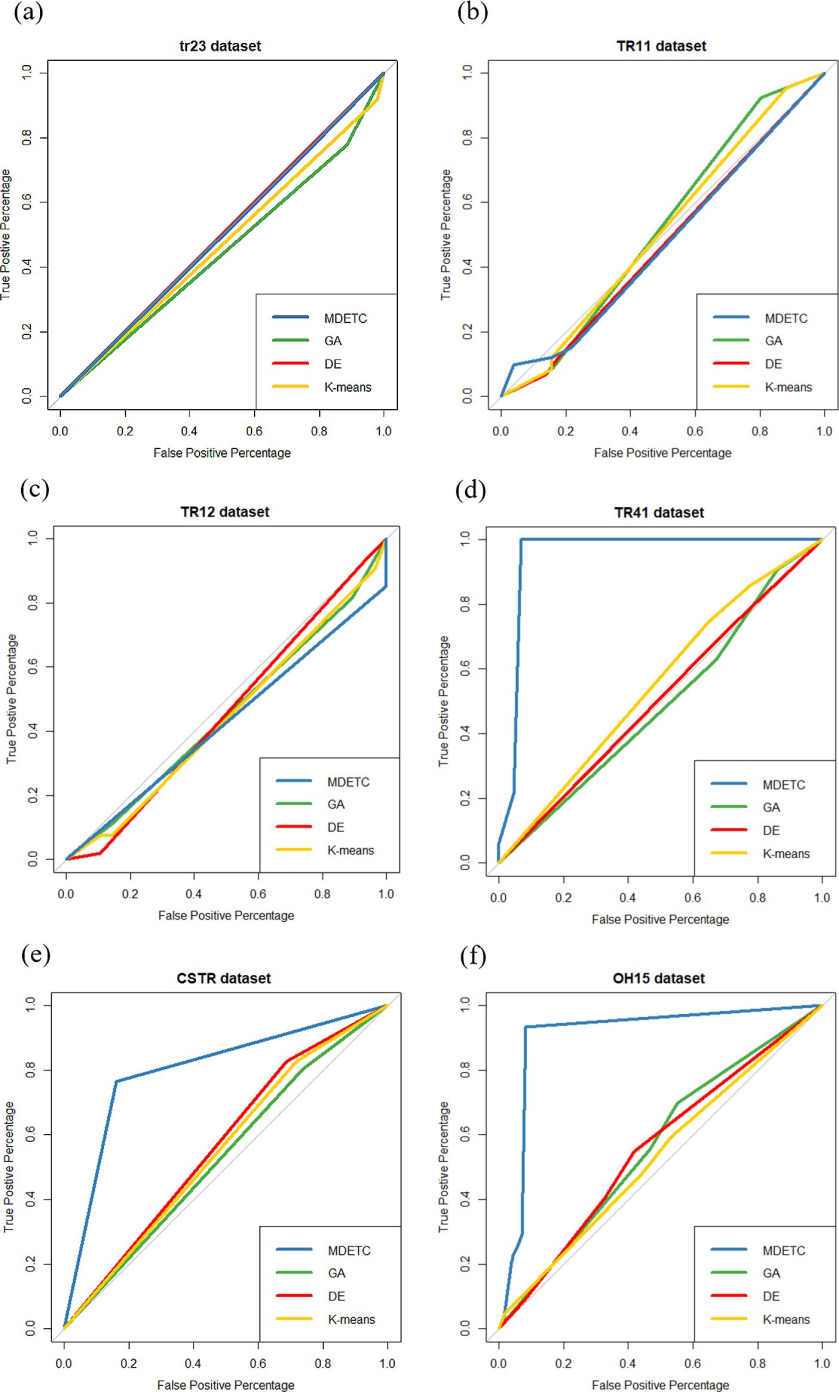
The ROC curves on (a) tr23, (b) tr11; (c) tr12; (d) tr41; (e) CSTR; (f) oh15 datasets.

Fig 5 shows the corresponding ROC curves obtained by the MDETC, K-means, DE and GA algorithms on the used datasets. The ROC curves demonstrate that MDETC produces excellent performance on tr41, CSTR, and oh15 datasets with better capability to distinguish between classes. Thus, MDETC obtained fair performance compared with the competing algorithms on tr23, tr11, and tr12 datasets.

Table 6 demonstrates the average results of the F-measure obtained by competing algorithms. The proposed MDETC achieved the best results on all datasets concerning the F-measure (i.e., tr23, tr11, tr12, tr41, CSTR, and oh15). The results show that the proposed MDETC algorithm has a higher F-measure compared with the competing algorithms, for example, the results of CSTR dataset indicates that MDETC obtained an F-measure value of 0.6908, whereas the F-measure results of K-means, DE, and Genetic Algorithm (GA) are 0.5008, 0.5429, and 0.5133, respectively. However, the F-measure result achieved by GA is close to MDETC on tr12 datasets.

**Table 6:** The comparison of F-measure values obtained by the MDETC, K-means, DE and GA algorithms.

Dataset	K-means	DE	GA	MDETC
tr23	0.5759	0.5791	0.5572	**0.6240**
tr11	0.5043	0.4398	0.4595	**0.5414**
tr12	0.3402	0.4114	0.4470	**0.4481**
tr41	0.4494	0.4030	0.3685	**0.6269**
CSTR	0.5008	0.5429	0.5133	**0.6908**
oh15	0.3709	0.2976	0.2788	**0.5895**

Moreover, the results in Table 6 are further analyzed using the rankings generated by Friedman’s test based on the F-measure, as shown in Table 7. Friedman’s test has shown that MDETC obtained a significant difference with a *p*-value of 0.00974 that is below the significance level (*α* = 0.05). The results confirm that MDETC obtained the best ranking based on the F-measure. The DE obtained the second-best rank, and then the K-means algorithm. Finally, GA achieved the worst rank.

**Table 7:** Friedman test ranking for MDETC, K-means, DE and GA algorithms based on the F-measure.

Algorithm	Ranking
MDETC	1
DE	2.833
K-means	2.833
GA	3.333

Moreover, the statistical difference between the control case (MDETC) and the other algorithms is detected using the Holm’s post-hoc procedure. Table 8 demonstrates the *p*-value achieved by Holm’s procedure, where the null hypothesis is rejected based on the achieved *p*-value that needs to be less than the adjusted value of α (α*/i*). The Holm’s procedure demonstrates that MDETC is statistically better than K-means, DE and GA.

**Table 8:** Comparison between MDETC, K-means, DE and GA algorithms using Holm’s post-hoc procedure based on the F-measure.

*i*	Algorithm	α/*i*	*p*-value of Holms	Null Hypothesis
1	DE	0.05/1 = 0.0500	0.013906	Rejected
2	K-means	0.05/2 = 0.0250	0.013906	Rejected
3	GA	0.05/3 = 0.0166	0.001745	Rejected

**Fig 6:**
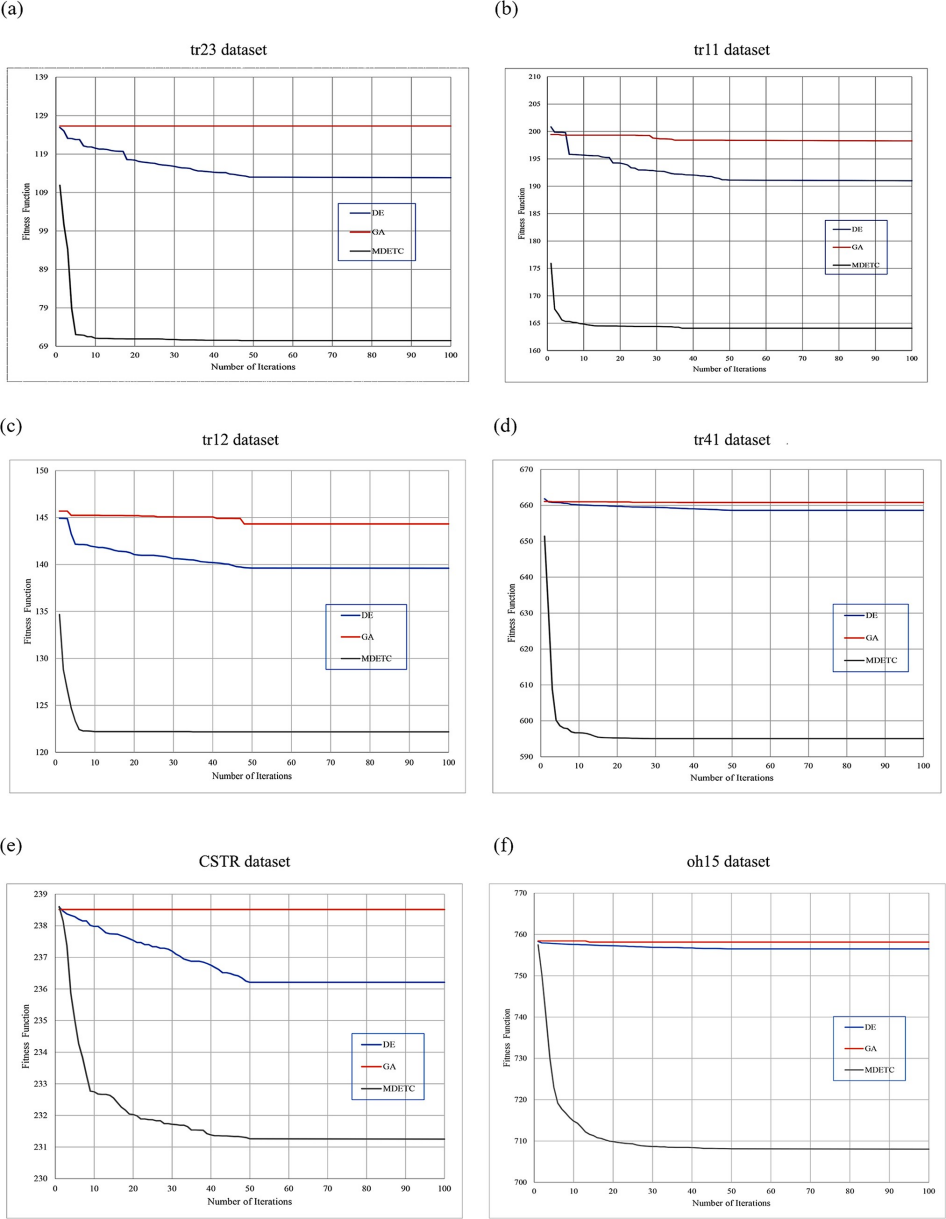
The convergence curves on (a) tr23, (b) tr11; (c) tr12; (d) tr41; (e) CSTR; (f) oh15 datasets.

Fig 6 shows the convergence curves on the employed datasets. The curves demonstrate that MDETC produces the best convergence performance on the six datasets with fast convergence in the initial iterations; next, convergence becomes slower. The proposed memetic steps improved efficiency by avoiding premature convergence. The DE obtained the second best convergence rate results, and GA obtained the worst results.

Table 9 shows the running time of a single iteration of the proposed MDETC, K-means, DE and GA algorithms on the related datasets to investigate the complexity of these algorithms. As presented in Table 9, The GA algorithm obtained the best results of the processing time on all datasets. Nevertheless, the MDETC requires less processing time compared to DE and K-means algorithms on the employed datasets except for the tr23 dataset. The K-means achieved the best third-best processing time on the entire datasets except for the tr12 dataset. The DE did not achieve any shorter running time on the test datasets except for the tr12 dataset. Consequently, the trade-off between the time-cost and the quality problem appeared, where hybrid metaheuristic methods, such as MDETC, can achieve optimal solutions in acceptable running time. On the other hand, the traditional metaheuristic algorithm does not promise to obtain the optimal solution and commonly can produce sub-optimal and good-quality solutions in shorter running time.

**Table 9:** Running time of MDETC, K-means, DE and GA algorithms.

Dataset	K-means	DE	GA	MDETC
tr23	0.301	0.517	**0.052**	0.362
tr11	1.001	1.211	**0.070**	0.831
tr12	0.784	0.770	**0.059**	0.533
tr41	1.211	2.907	**0.192**	2.002
CSTR	0.201	0.246	**0.021**	0.171
oh15	1.109	1.343	**0.069**	0.918

### Comparison between MDETC and state of the art

The performance of MDETC is compared with the state of the art algorithms, such as the hybrid krill herd algorithm (MMKHA) [10], krill herd algorithm (KH) [10], particle swarm optimization (PSO) [49], Hybrid Harmony Search (HS) [34]. As presented in Table 10, the F-measure achieved by MDETC is better than competing algorithms. The MDETC obtained the optimum F-measure on the tr23, tr11, tr41, CSTR, and oh15 datasets. The MMKHA algorithm obtained the optimum F-measure on the tr12 dataset and scored the second-best result on the remaining datasets. The results presented in Table 10 reveals that MDETC achieved consistent performance across all datasets using the F-measure.

**Table 10:** F-measure comparison between MDETC and the state of art algorithms.

Dataset	HS	KH	PSO	MMKHA	MDETC
tr23	0.4021	0.4004	0.3565	0.4214	**0.6240**
tr11	0.4095	0.4138	0.4380	0.5164	**0.5414**
tr12	0.4526	0.5019	0.4708	**0.5624**	0.4481
tr41	0.4392	0.4272	0.4471	0.5241	**0.6269**
CSTR	0.5268	0.4847	0.5090	0.6055	**0.6908**
oh15	0.4185	0.4840	0.4471	0.5278	**0.5895**

Additionally, the results in Table 10 are further analyzed using the rankings generated by Friedman’s test based on the F-measure, as shown in Table 11. The test has shown that MDETC obtained a significant difference with a *p*-value of 0.01302 that is below the significance level (*α* = 0.05). The results confirm that MDETC obtained the best ranking based on the F-measure. The MMKHA algorithm attained the second-best rank, and the PSO scored the third rank, then the KH. Finally, HS achieved the worst rank. The rankings presented in Table 11 show that MDETC performance based on the F-measure is consistent when compared with the state of art algorithms.

**Table 11:** Friedman test ranking for MDETC and the state of art algorithms based on the F-measure.

Algorithm	Ranking
MDETC	1.6666
MMKHA	1.8333
PSO	3.6666
KH	3.8333
HS	4.0

Moreover, Table 12 shows the *p*-value of MDETC and the state of art algorithms using Holm’s post-hoc procedure, where the null hypothesis is rejected based on the achieved *p*-value that needs to be less than the adjusted value of α (α*/i*). The Holm’s procedure shown in Table 12 demonstrates that MDETC is statistically better than PSO, KH, and HS. Thus, MDETC is not significantly different from the MMKHA algorithm. However, the results presented in Table 10 confirm that the MDETC algorithm outperformed the MMKHA based on the tested datasets.

**Table 12:** Comparison between MDETC and the state of art algorithms using Holm’s procedure based on the F-measure.

*i*	Algorithm	α/*i*	*p*-value of Holms	Null Hypothesis
1	MMKHA	0.05/1 = 0.0500	0.85513	Not rejected
2	PSO	0.05/2 = 0.0250	0.02445	Rejected
3	KH	0.05/3 = 0.0166	0.01762	Rejected
4	HS	0.05/4 = 0.0125	0.01058	Rejected

## Conclusions and future work

This work proposed an MDETC algorithm for addressing the text clustering problem. The combination of DE and Memetic algorithms intends to achieve a better balance between exploration and exploitation. The algorithm introduced a DE mutation operator that is hybridized within the Memetic algorithm. To prove the effectiveness of the introduced algorithm, six standard text clustering benchmark datasets (i.e. the Laboratory of Computational Intelligence (LABIC)) employed to assess the presented algorithm. The Experimental results confirmed that the introduced MDETC algorithm obtained consistent performance compared to the state of art algorithms concerning the AUC metric and F-measure validity measures. These results revealed that the proposed MDETC has achieved a better balance between exploration and exploitation and improved the performance of the Memetic algorithms to solve the text clustering problem. The MDETC algorithm obtained the optimum results of the F-measure on tr23 (62.4%), tr11 (54.14%), tr41 (62.69%), CSTR (69.08%), and oh15 (58.95%) datasets. Furthermore, the future work will concentrate on incorporating different validity measures when employed within the multi-objective metaheuristic algorithms.

## Supporting information

S1 File (DOCX)

## Abbreviations

ABC: Artificial bee colony
ACO: Ant colony optimization
CS: Cuckoo search
DE: Differential Evolution algorithm
FA: irefly algorithm
GA: Genetic Algorithm
HS: Harmony search algorithm
KHA: Krill herd algorithm
MA: Memetic Algorithm
MDETC: Memetic differential evolution for solving text clustering problems
MMKHA: Hybrid krill herd algorithm
PSO: Particle Swarm Optimizer
TC: Text clustering
TD: Text document
C: Set of all Clusters
Cr: Crossover constant
D: Set of all text documents
d: Text document
F: DE Differentiation constant
i: Text document number *i*
K: Number of all clusters
MGI: Max generation without improvement
n: Number of all text documents in set *D*
nl: Number of documents in cluster l
Pool_Size: Recombination mating pool size
Pop_Size: The population size constant
t: Number of unique terms that exist in the entire documents
Tour_Size: Tournament selection size
wi,j: Weight of term j in document *i*
Zl: The centroid of cluster *l*
ABC: Artificial bee colony
ACO: Ant colony optimization
CS: Cuckoo search
DE: Differential Evolution algorithm
FA: Firefly algorithm
GA: Genetic Algorithm
HS: Harmony search algorithm
KHA: Krill herd algorithm
MA: Memetic Algorithm
MDETC: Memetic differential evolution for solving text clustering problems
MMKHA: Hybrid krill herd algorithm
PSO: Particle Swarm Optimizer
TC: Text clustering
TD: Text document
C: Set of all Clusters
Cr: Crossover constant
D: Set of all text documents
d: Text document
F: DE Differentiation constant
i: Text document number i
K: Number of all clusters
MGI: Max generation without improvement
n: Number of all text documents in set D
nl: Number of documents in cluster l
Pool_Size: Recombination mating pool size
Pop_Size: The population size constant
t: Number of unique terms that exist in the entire documents
Tour_Size: Tournament selection size
wi,j: Weight of term j in document i
Zl: The centroid of cluster l

## References

[1] Wu W, Xiong H, Shekhar S. Clustering and Information Retrieval. 1st ed Springer Science & Business Media; 2013 10.1007/978-1-4613-0227-8

[2] Abbasi AA, Younis M. A survey on clustering algorithms for wireless sensor networks. Comput Commun. 2007;30: 2826–2841. 10.1016/j.comcom.2007.05.024

[3] Gupta Twinkle, Kumar Dharmender. Optimization of Clustering Problem Using Population Based Artificial Bee Colony Algorithm: A Review. Int J Adv Res Comput Sci Softw Eng. 2014;4: 491–502.

[4] Shokouhifar M, Jalali A. Optimized sugeno fuzzy clustering algorithm for wireless sensor networks. Eng Appl Artif Intell. 2017;60: 16–25. doi: https://doi.org/10.1016/j.engappai.2017.01.007

[5] Kang J, Zhang W. Combination of Fuzzy C-Means and Particle Swarm Optimization for Text Document Clustering In: Xie A, Huang X, editors. Advances in Electrical Engineering and Automation. Berlin, Heidelberg: Springer Berlin Heidelberg; 2012 pp. 247–252.

[6] Aggarwal CC, Zhai C. A Survey of Text Clustering Algorithms Mining Text Data. Boston, MA: Springer US; 2012 pp. 77–128. 10.1007/978-1-4614-3223-4_4

[7] Lu Y, Zhang P, Liu J, Li J, Deng S. Health-Related Hot Topic Detection in Online Communities Using Text Clustering. PLoS One. Public Library of Science; 2013;8: 1–9. 10.1371/journal.pone.0056221 23457530 PMC3574139

[8] Song W, Qiao Y, Park SC, Qian X. A hybrid evolutionary computation approach with its application for optimizing text document clustering. Expert Syst Appl. 2015;42: 2517–2524. 10.1016/j.eswa.2014.11.003

[9] Abualigah LM, Khader AT, Al-Betar MA. Multi-objectives-based text clustering technique using K-mean algorithm. Proceeding of the 7th International Conference on Computer Science and Information Technology (CSIT). 2016. pp. 1–6. 10.1109/CSIT.2016.7549464

[10] Abualigah LM, Khader AT, Hanandeh ES. Hybrid clustering analysis using improved krill herd algorithm. Appl Intell. 2018;48: 4047–4071. 10.1007/s10489-018-1190-6

[11] Abualigah LM, Khader AT, Hanandeh ES. A new feature selection method to improve the document clustering using particle swarm optimization algorithm. J Comput Sci. 2018;25: 456–466. 10.1016/j.jocs.2017.07.018

[12] Wu TH, Yeh JY, Lee YM. A particle swarm optimization approach with refinement procedure for nurse rostering problem. Comput Oper Res. Elsevier; 2015;54: 52–63. 10.1016/j.cor.2014.08.016

[13] Rodriguez MZ, Comin CH, Casanova D, Bruno OM, Amancio DR, Costa L da F, et al Clustering algorithms: A comparative approach. PLoS One. 2019;14: 1–34. 10.1371/journal.pone.0210236 30645617 PMC6333366

[14] Bouyer A, Hatamlou A. An efficient hybrid clustering method based on improved cuckoo optimization and modified particle swarm optimization algorithms. Appl Soft Comput J. Elsevier B.V.; 2018;67: 172–182. 10.1016/j.asoc.2018.03.011

[15] Jaradat G, Ayob M, Almarashdeh I. The effect of elite pool in hybrid population-based meta-heuristics for solving combinatorial optimization problems. Appl Soft Comput J. Elsevier B.V.; 2016;44: 45–56. 10.1016/j.asoc.2016.01.002

[16] Yassen ET, Ayob M, Zakree M, Nazri A. The effects of hybridizing local search algorithms with harmony search for the vehicle routing problem with time windows. J Theor Appl Inf Technol. 2015;73: 43–58.

[17] Yassen ET, Ayob M, Nazri MZA, Sabar NR. An adaptive hybrid algorithm for vehicle routing problems with time windows. Comput Ind Eng. 2017;113: 382–391. 10.1016/j.cie.2017.09.034

[18] Ramadan RM, Gasser SM, El-Mahallawy MS, Hammad K, El Bakly AM. A memetic optimization algorithm for multi-constrained multicast routing in ad hoc networks. PLoS One. 2018;13: 1–17. 10.1371/journal.pone.0193142 29509760 PMC5839550

[19] Sabar NR, Ayob M, Kendall G. A Hybrid of Differential Evolution and Simulated Annealing Algorithms for the Capacitated Arc Routing Problems. Proceedings of the 6th Multidisciplinary International Conference on Scheduling:Theory and Applications. Gent, Belgium; 2013. pp. 549–554.

[20] Mustafa H, Ayob M, Nazri MZA, Abu-Taleb S. Multi-objectives memetic discrete differential evolution algorithm for solving the container pre-marshalling problem. J Inf Commun Technol. 2019;18: 77–96.

[21] Mustafa HMJ, Ayob M, Nazri MZA, Kendall G. An improved adaptive memetic differential evolution optimization algorithms for data clustering problems. PLoS One. 2019;14: e0216906 10.1371/journal.pone.0216906 31137034 PMC6538400

[22] Song W, Li CH, Park SC. Genetic algorithm for text clustering using ontology and evaluating the validity of various semantic similarity measures. Expert Syst Appl. 2009;36: 9095–9104. 10.1016/j.eswa.2008.12.046

[23] Akter R, Chung Y. An Evolutionary Approach for Document Clustering. IERI Procedia. 2013;4: 370–375. 10.1016/j.ieri.2013.11.053

[24] Cui X, Potok TE, Palathingal P. Document clustering using particle swarm optimization. Proceedings 2005 IEEE Swarm Intelligence Symposium, 2005 SIS 2005. 2005. pp. 185–191. 10.1109/SIS.2005.1501621

[25] Song W, Ma W, Qiao Y. Particle swarm optimization algorithm with environmental factors for clustering analysis. Soft Comput. 2017;21: 283–293. 10.1007/s00500-014-1458-7

[26] Amiri E, Mahmoudi S. Efficient protocol for data clustering by fuzzy Cuckoo Optimization Algorithm. Appl Soft Comput. 2016;41: 15–21. 10.1016/j.asoc.2015.12.008

[27] Nagarajan E, Saritha K, MadhuGayathri G. Document clustering using ant colony algorithm. Proceeding of the International Conference on Big Data Analytics and Computational Intelligence (ICBDAC). 2017. pp. 459–463. 10.1109/ICBDACI.2017.8070884

[28] Bharti KK, Singh PK. Chaotic Artificial Bee Colony for Text Clustering. Proceeding of the 4th International Conference of Emerging Applications of Information Technology. 2014. pp. 337–343. 10.1109/EAIT.2014.48

[29] Bharti KK, Singh PK. Chaotic gradient artificial bee colony for text clustering. Soft Comput. 2016;20: 1113–1126. 10.1007/s00500-014-1571-7

[30] Mohammed AJ, Yusof Y, Husni H. GF-CLUST: A nature-inspired algorithm for automatic text clustering. J Inf Commun Technol. 2016;15: 57–81.

[31] Devi SS, Shanmugam A, Prabha ED. A Proficient Method for Text Clustering Using Harmony Search Method. Int J Sci Res Sci Eng Technol. 2015;1: 145–150.

[32] Wang H, Xu Z, Pedrycz W. An overview on the roles of fuzzy set techniques in big data processing: Trends, challenges and opportunities. Knowledge-Based Syst. 2017;118: 15–30. 10.1016/j.knosys.2016.11.008

[33] Zaw MM, Mon EE. Web Document Clustering by Using PSO-Based Cuckoo Search Clustering Algorithm. Recent Advances in Swarm Intelligence and Evolutionary Computation. Springer International Publishing; 2015 pp. 263–281. 10.1007/978-3-319-13826-8_14

[34] Forsati R, Mahdavi M, Shamsfard M, Meybodi MR. Efficient stochastic algorithms for document clustering. Inf Sci (Ny). 2013;220: 269–291. 10.1016/j.ins.2012.07.025

[35] Manikandan P, Selvarajan S. Data Clustering Using Cuckoo Search Algorithm (CSA). In: Babu B V, Nagar A, Deep K, Pant M, Bansal JC, Ray K, et al., editors. Proceedings of the Second International Conference on Soft Computing for Problem Solving (SocProS 2012), December 28–30, 2012. New Delhi: Springer India; 2014. pp. 1275–1283.

[36] Saida IB, Nadjet K, Omar B. A New Algorithm for Data Clustering Based on Cuckoo Search Optimization In: Pan J-S, Krömer P, Snášel V, editors. Genetic and Evolutionary Computing. Cham: Springer International Publishing; 2014 pp. 55–64. 10.1007/978-3-319-01796-9_6

[37] Hassanzadeh T, Meybodi MR. A new hybrid approach for data clustering using firefly algorithm and K-means. Proceeding of the 16th CSI International Symposium on Artificial Intelligence and Signal Processing. 2012. pp. 7–11. 10.1109/AISP.2012.6313708

[38] Forsati R, Keikha A, Shamsfard M. An improved bee colony optimization algorithm with an application to document clustering. Neurocomputing. 2015;159: 9–26. 10.1016/j.neucom.2015.02.048

[39] Kheng CW, Chong SY, Lim MH. Centroid-based memetic algorithm-adaptive Lamarckian and Baldwinian learning. Int J Syst Sci. 2012;43: 1193–1216. 10.1080/00207721.2011.617526

[40] Sörensen K, Sevaux M. MA|PM: Memetic algorithms with population management. Comput Oper Res. 2006;33: 1214–1225. 10.1016/j.cor.2004.09.011

[41] Zhou A, Qu B-Y, Li H, Zhao S-Z, Suganthan PN, Zhang Q. Multiobjective evolutionary algorithms: A survey of the state of the art. Swarm Evol Comput. 2011;1: 32–49. 10.1016/j.swevo.2011.03.001

[42] Das S, Mullick SS, Suganthan PN. Recent advances in differential evolution–An updated survey. Swarm Evol Comput. 2016;27: 1–30. 10.1016/j.swevo.2016.01.004

[43] Ali MZ, Awad NH, Suganthan PN, Reynolds RG. An Adaptive Multipopulation Differential Evolution with Dynamic Population Reduction. IEEE Trans Cybern. 2017;47: 2768–2779. 10.1109/TCYB.2016.2617301 28113798

[44] Neri F, Tirronen V. On Memetic Differential Evolution frameworks: A study of advantages and limitations in hybridization. 2008 IEEE Congress on Evolutionary Computation, CEC 2008. 2008. pp. 2135–2142. 10.1109/CEC.2008.4631082

[45] Neri F, Cotta C, Moscato P. Handbook of Memetic Algorithms Studies in Computational Intelligence,Volume 379 Springer; 2012: 370 10.1007/978-3-642-23247-3

[46] Sivanandam SN, Deepa SN. Introduction to genetic algorithms 1st ed Introduction to Genetic Algorithms. Springer-Verlag Berlin Heidelberg; 2008 10.1007/978-3-540-73190-0

[47] Miller BL, Goldberg DE. Genetic Algorithms Tournament Selection and the Effects of Noise. Complex Syst. 1995;9: 193–212.

[48] LABIC. Laboratory of Computational Intelligence (LABIC) [Internet]. 2019. Available: http://sites.labic.icmc.usp.br/text_collections/

[49] Karol S, Mangat V. Evaluation of text document clustering approach based on particle swarm optimization. Cent Eur J Comput Sci. 2013;3: 69–90. 10.2478/s13537-013-0104-2

