# Supplementary material for: Solving text clustering problem using a memetic differential evolution algorithm
Source: PLoS One. 2020 Jun 11;15(6):e0232816. doi: 10.1371/journal.pone.0232816 (PMC7289410; doi:10.1371/journal.pone.0232816)
Supplement: S1 File — (DOCX) [file pone.0232816.s001.docx]

Data are available from the Laboratory of Computational Intelligence (LABIC) (URLs: <http://sites.labic.icmc.usp.br/text_collections/oh15.arff.zip>;

<http://sites.labic.icmc.usp.br/text_collections/tr11.arff.zip>;

<http://sites.labic.icmc.usp.br/text_collections/tr12.arff.zip>;

<http://sites.labic.icmc.usp.br/text_collections/tr41.arff.zip>;

<http://sites.labic.icmc.usp.br/text_collections/tr23.arff.zip>;

<http://sites.labic.icmc.usp.br/text_collections/CSTR.arff.zip> ).
