# Supplementary material for: Solving text clustering problem using a memetic differential evolution algorithm
Source: PLoS One. 2020 Jun 11;15(6):e0232816. doi: 10.1371/journal.pone.0232816 (PMC7289410; doi:10.1371/journal.pone.0232816)
Supplement: Attachment — Submitted filename: Response to Reviewers.docx [file pone.0232816.s002.docx]

Dear editor of Plos One journal,

Thank you for the valuable feedback. We have added some sentences/paragraphs in order to response the comments from the reviewers. The following table summarizes the updates that have been made to the paper as a response to the reviewers’ comments. Moreover, additional statistical analysis is added in the experimental results section to fulfill the journal requirement that have been addressed by the reviewers.

| **#** | **Reviewer #1 comment** | **Author response** |
| --- | --- | --- |
| 1 | The motivations for this study need to be made clearer | The motivation is modified in the introduction section, and cleared in the closing paragraph of the related work. |
| 2 | Figures must be in a good quality with high resolution | All figures is modified with better resolution according to the journal figures requirements. |
| 3 | The manuscript is poorly written. Authors really need to improve their written communications skills in English | The manuscript grammar is rechecked and proofread. |
| 4 | All references must be revised. Issue numbers and volume numbers are missed from most of references | All references mentioned in the comment are revised accordingly. |
| 5 | Conclusions should address whether or not they achieved the objective of the study. | The conclusion section has been modified to include the achievement of the objective of the study. |
| **#** | **Reviewer #2 comment** | **Author response** |
| 1 | There are multiple grammatical and editorial errors in the text | The manuscript grammar is rechecked and proofread. |
| 2 | There seem to be unnecessary amount of citations to the works performed by one other group. Namely, citations 47,10,11 and then 12 and 13 | References 11, 12, and 47 is removed.  The remaining references are kept since they represent different works in text clustering such as multiobjective optimization, unsupervised feature selection, and Hybrid krill herd algorithm approaches. |
| 3 | A disturbing amount of abbreviations and unnecessary nomenclature has been used that is not conductive to the cohesion of the report.  You will need to do a better job defining the two different abbreviations TC and TD in the manuscript. | All abbreviations have been revised accordingly. The TD and TC are discussion is modified in the introduction section. |
| 4 | In the section "population initialization phase" the authors refer to equation (4) for computing cluster centroids. I believe they intended to refer to equation (6). | The equation number is corrected to be equation (6) (calculation of cluster centroids) |
| 5 | The authors are using F-value or F-measure as their evaluation metric, I think it is a good practice to include the metric definition in the manuscript. Moreover, when referring to this metric they cite a 2017 paper. | The unnecessary citation is removed since its only indicates that this measure have been used in similar studies. The equations and discussion of F-measure are added in the experimental setup section. |
| 6 | In the section "Parameter setting used in the study": how did the authors come to this setting? Did they perform a parameter study? Or maybe cross-validation? | It is based on an experimental basis and the drawing on previous work from the scientific literature [3]*. This statement is added to the experimental setup section. |
| 7 | Number of terms in documents (table 1) seem to be very low. For example 1700 unique words in a dataset of 299 documents (CSTR dataset). Or 3000 words in 900 documents. Maybe this numbers are after stemming and stop-word removal, but how are those steps performed? I would recommend, if not within the main manuscript, at least provide a supplementary material section where you describe this detailed methodology. | These datasets are standard benchmark text documents datasets that already pre-processed, the description is added in the experimental setup section. The detailed steps of the pre-processing are elaborated in background section (text clustering problem). |
| 8 | The authors are using tf-idf and vector space model for their document representation. Why not using a more elaborate word-representation such as Word2Vec or GloVe. Is there a benefit in using tf-idf and not more modern representations? Needs to be explained. | The following are the reasons to adopt the *tf-idf* in our algorithm:   1. The TF/IDF is commonly used by TC algorithms, where the frequent terms will be a good indicator for a certain topic [1] [2]* 2. The standard text document clustering datasets are represented (pre-processed) by *tf-idf* term frequency.   More discussion is added to the Text clustering problem section. And also more discussion is added (as in comment No. 7). |

*References.

1. Aggarwal CC, Reddy CK. Data Custering Algorithms and Applications. 1st ed. Taylor & Francis Group, LLC; 2013.

2. Cui X, Potok TE, Palathingal P. Document clustering using particle swarm optimization. Proc 2005 IEEE Swarm Intell Symp 2005 SIS 2005. 2005; 185–191. doi:10.1109/SIS.2005.1501621

3. Mustafa HMJ, Ayob M, Nazri MZA, Kendall G. An improved adaptive memetic differential evolution optimization algorithms for data clustering problems. PLoS One. 2019;14(5): e0216906. doi:10.1371/journal.pone.0216906

Sincerely,
Hossam M. J. Mustafa, PhD Candidate,
Corresponding Author,
Faculty of Information Science and Technology (FTSM),
National University of Malaysia (UKM),
43000 Bangi, Selangor, Malaysia
