# Supplementary material for: Solving text clustering problem using a memetic differential evolution algorithm
Source: PLoS One. 2020 Jun 11;15(6):e0232816. doi: 10.1371/journal.pone.0232816 (PMC7289410; doi:10.1371/journal.pone.0232816)
Supplement: Attachment — Submitted filename: Response to Reviewers.docx [file pone.0232816.s003.docx]

Dear editor of Plos One journal,

Thank you for the valuable feedback. We have added some sentences/paragraphs to respond to the comments from the reviewers. The following table summarizes the updates that have been made to the paper as a response to the reviewers’ comments. Moreover, additional statistical analysis is added in the experimental results section to fulfill the journal requirement that has been addressed by the reviewers.

| **#** | **Reviewer #3 comment** | **Author response** |
| --- | --- | --- |
| 1 | I suggest all abbreviations of algorithms such as KH, CS, PSO, etc. be removed, they are not necessary and still excessive, making the manuscript hard to follow. | The abbreviations are used within the related work and the comparison with the state of art sections. We defined these abbreviations whenever are used, then the abbreviations are used due to difficulties of using the full name of the algorithms within the figures and tables, for example, hybrid krill herd algorithm (MMKHA). |
| 2 | The abstract needs to be improved as well | We revised the abstract and made proofreading. |
| **#** | **Reviewer #4 comment** | **Author response** |
| 1 | The English could still be improved | Proofreading is made. |
| **#** | **Reviewer #5 comment** | **Author response** |
| 1 | Line 390 **(460 in the new manuscript)**: in the comparative analysis section: Why is the work from Ref 23 not included here? What is the difference between this work and the work presented in Ref 23? | Ref 23 **(21 in the new manuscript)** uses different objective functions and local search heuristic. In Ref 23, the datasets used to evaluate the algorithm is low dimension benchmark datasets that are collected from many domains. More discussion about this issue can be found in the related work section (las paragraphs line .. ). The text clustering datasets (as mentioned in the background section) use different kind of data (tf/idf) and requires different objective function, evolutionary steps, and local search to be proposed. |
| 2 | Line 340 **(403 in the new manuscript)**: The use of F-measure sounds good, but it is logical to present the full spectrum of evaluation metrics before narrowing down to one. For instance, the reader would be interested to know the AUC, in addition to the F-measure. | The AUC metric and ROC are included in the study. A discussion about these evaluation metrics is added to the experiment setup section. Besides, the ROC curve (figure 5) and the results of the AUC metric (tables 3-5) are added and compared using the statistical analysis in the experimental results and discussion section. The abstract, introduction, and conclusion are revised accordingly. |
| 3 | Line 233 to 235 **(235 in the new manuscript)**: The study failed to explain the limitation of the existing algorithm. The use of overly broad terminology does not account for the limitation. Furthermore, the study failed to state the limitation of MA and DE for which an extended is considered important. I think these are missing steps that can improve the paper | A paragraph is added before the comment to elaborate the need to hybridize between DE and MA with three benefits ( see appendix A) |
| 4 | Reference to the Related works: The language is really bad. Another round of proofreading would be required. One more thing, the sequence of the reference can be adjusted in ascending order. It is presented in a scattered manner. This does not aid reading. Overall, the manuscript failed in readability due to poor grammar and sentence structure | We reorganized and revised the related work section accordingly. The sequence of references is adjusted. The related work is grouped according to the metaheuristic algorithm or approach used. |

The following table summarizes the updates that have been made to the paper as a response to the editor's comments concerning the Journal requirements.

| **#** | **Editor comment** | **Author response** |
| --- | --- | --- |
| 1 | One of the reviewers raises their concern where the similarity between this paper with other published paper entitled "An improved adaptive memetic differential evolution optimization algorithms for data clustering problems" under PLoS One as well. Please emphasize the difference between these two papers and highlight your contribution explicitly | Please note that the algorithm proposed in this manuscript is an enhanced algorithm of the previously published paper by PLOS ONE “An improved adaptive memetic differential evolution optimization algorithms for data clustering problems”. The proposed algorithm uses different memetic stages (such as improvement stage and restart stage) and employs different objective functions to solve high dimensional text clustering benchmark datasets (which are real-life UCI datasets in the previous paper). Additionally, the literature related to the proposed algorithm is different from the previously mentioned paper. This issue is discussed in the related work (line 133-145) as a response to the comment. |

Sincerely,
Hossam M. J. Mustafa,
Corresponding Author,
Faculty of Information Science and Technology (FTSM),
National University of Malaysia (UKM),
43000 Bangi, Selangor, Malaysia

**Appendices**

**Appendix A**

The MA utilized other optimization algorithms by employing them inside the framework [41]. For example, metaheuristic algorithms such as Differential Evolution has shown better mutation performance [42] with appropriate parameter settings and mutation strategies. The combination of DE within the MA can offer three benefits: Firstly, the offspring’s quality produced by evolutionary algorithms such as MA can be improved by implementing several search methods in the optimization search process. An example of these search methods is the DE mutation, which can be employed to generate better quality individuals [43]. Secondly, premature convergence and stagnation can be minimized when employing a DE algorithm by balancing exploitation and exploration, which can be achieved by utilizing several mutation strategies [19]. Thirdly, the DE population can stagnate that occurs when the offspring are less fit than their parents over a given number of iterations. To address this, the DE’s performance can be enhanced by employing a convenient hybridization including local search algorithms within the MA framework [43,44].
